# PARP inhibitor augments anti-tumor efficacy of DNMT inhibitor by inducing senescence in cholangiocarcinoma

**DOI:** 10.7150/ijbs.110947

**Published:** 2025-05-27

**Authors:** Peili Wang, Rong Xiao, Jianfeng Chen, Peiyong Guan, Hong Lee Heng, Lizhen Liu, Yali Wang, Xian Zeng, Guixiang Zhong, Jing Hao, Jiuping Gao, Jason Yongsheng Chan, Simona Dima, Choon Kiat Ong, Bin Tean Teh, Mei Li, Jing Han Hong, Jing Tan

**Affiliations:** 1 Sun Yat-sen University Cancer Center, State Key Laboratory of Oncology in South China, Guangdong Provincial Clinical Research Center for Cancer, 510060, Guangzhou, Guangdong, P. R. China; 2 Genome Institute of Singapore A*STAR, Singapore 138672, Singapore; 3 Laboratory of Cancer Epigenome, Division of Medical Science, National Cancer Center, Singapore, Singapore; 4 Guangdong Provincial People's Hospital, Guangdong Academy of Medical Sciences, School of Medicine, South China University of Technology Guangzhou, China; 5 Department of Oncology, The First Affiliated Hospital, Sun Yat-sen University, Guangzhou, Guangdong Province, 510080, China.; 6 Division of Medical Oncology, National Cancer Centre Singapore, Singapore; 7 Center of Excellence for Translational Medicine, Fundeni Clinical Institute, Bucharest, Romania;; 8 University of Medicine and Pharmacy "Carol Davila", Bucharest, Romania.; 9 Lymphoma Translational Research Laboratory, National Cancer Centre Singapore, Singapore; 10 Cancer and Stem Cell Biology Program, Duke-NUS Medical School, 169857, Singapore; 11 Hainan Academy of Medical Science, Hainan Medical University, Haikou, PR China

**Keywords:** cholangiocarcinoma, DNMT inhibitor, PARP inhibitor, cellular senescence

## Abstract

Cholangiocarcinoma (CCA) is an aggressive, heterogeneous malignancy with limited effective treatment options. One of the key epigenetic dysregulations in CCA is aberrant DNA hypermethylation, suggesting that targeted DNA methylation is a promising therapeutic strategy for this disease. However, there is still limited information on how effective DNA demethylating agents are in the treatment of CCA in the clinical setting, and further studies are urgently needed to evaluate their potential benefits. Here, we established four patient-derived CCA cell lines and demonstrated that the DNA methyltransferase (DMNT) inhibitors decitabine and azacitidine had minimal effects on inhibiting CCA proliferation. A combinatorial drug screen identified PARP inhibitors as sensitizers that synergistically enhanced the antitumor effects of decitabine. The combination of DNMT inhibitors and PARP inhibitors therapeutically inhibited the growth of CCA cancers in multiple* in vitro* cancer cell lines and organoid models, as well as *in vivo* cell line-derived xenografts, patient-derived xenograft models, and CCA in mice induced by hydrodynamic tail vein injection. Mechanistically, transcriptomic profiling analysis showed that combination treatment activated the inflammatory signaling pathway and suppressed the cell cycle-related pathways in CCA. In addition, the combination synergistically induced DNA damage and cellular senescence of CCA cancer cells. Together, our study provides a preclinical proof-of-concept for the use of DNMT inhibitors in combination with PARP inhibitors as a novel therapeutic strategy and potentially optimizes current clinical practice in the treatment of CCA.

## Introduction

Cholangiocarcinoma (CCA) is a heterogeneous and aggressive malignant biliary tumor that accounts for approximately 15% of all primary liver cancers, is highly prevalent in Asia, and correlates with HBV and liver fluke infections 1. Surgery with chemoradiotherapy is the first-line treatment for patients with CCA, but most patients inevitably develop refractory disease or relapse with 5-year survival rate at less than 10% 2. Recent comprehensive molecular characterizations have provided new therapeutic targets for the treatment of CCA 3-5. For example, previous studies have divided CCA into four subtypes based on genomic mutations and revealed the molecular characteristics of CCA by integrating multi-omics data 6, suggesting that activating fusions of *FGFR* and mutations of *IDH1/2* can be used as new therapeutic targets. Indeed, targeted treatments were approved for a subset of patients who harbored *IDH1/2*-activating mutations or *FGFR2* fusions or rearrangements 7-9. However, these targeted drugs are only effective in a small number of patients harboring these genetic alterations. Therefore, there is an urgent need to develop novel therapeutic strategies for patients with CCA.

Epigenetic dysregulation is a hallmark feature of cancer, in which changes in gene expression contribute to tumorigenesis. Increasing evidence suggests that epigenetic alterations are associated with the carcinogenesis of CCA 4, 10-12. For example, many studies have identified high-frequency mutations of histone-modifying enzymes in CCA, such as* ARID1A*, *BAP1* and *PBRM1*. These mutations mediate epigenetic reprogramming and then alter the expression of oncogenes and tumor suppressor genes, thereby driving the initiation and development of CCA. In addition, Hong et al. reported that three molecular subtypes by enhancer profiling could provide potential targets for precision treatment of CCA 13. Furthermore, DNA hypermethylation has been detected at CpG islands or CpG shores depending on the etiology of CCA 6. DNA methyltransferases (DNMTs), which mediate DNA methylation, were upregulated in CCA 14, suggesting that DNA hypermethylation may be an attractive target for CCA treatment.

Aberrant hypermethylation of DNA can be reversed by targeting DNMTs. Azacitidine and decitabine are commonly used as DNA methyltransferase inhibitors (DNMTis) in the clinic. These are cytidine analogues that are incorporated into the DNA as a base and targeted for methylation, which lead to the covalent entrapment of the maintenance methylation enzyme DNMTs 15. As a result, they promote the proteasomal degradation of DNMTs, resulting in widespread methylation changes. Azacitidine and decitabine are widely used in numerous clinical trials for both hematological malignancies and solid tumors 16, which have been shown to provide benefits over conventional chemotherapy for patients with hematological malignancies or solid tumors 17. Unfortunately, these drugs were used at high doses or close to the maximum tolerated dose, similar for the use of cytotoxic chemotherapy agents 18, 19. As a result, combination therapy strategies as well as next-generation DNA methylation inhibitors are currently developed to overcome this challenge. To date, combinations of DNMTis with HDAC inhibitors, immunotherapy and cytotoxic chemotherapy have shown early clinical success. For example, in combination with immunotherapy, improved response has been facilitated by activating immune signaling through removal of methylation from promoter regions of silenced endogenous retroviruses 20, 21. These studies suggest that DNMTis in combination with other therapies may represent a potential strategy for patients with CCA.

In this study, we demonstrated the moderate anti-tumor effects of the demethylating agents decitabine and azacitidine in CCA cancer cells. Through drug library screening, we identified that PARP inhibitors could synergistically potentiate the efficacy of DNMTis in multiple preclinical CCA models. We also demonstrated that combination treatment increased DNA damage and caused cellular senescence in CCA. Our finding provides a potential therapeutic strategy to optimize current clinical practice in the treatment of CCA.

## Materials and Methods

### Clinical samples, cell cultures and reagents

EGI-1 was grown in DMEM (Invitrogen). HUCCT1, TFK-1 and QBC939 were grown in RPMI1640 (Invitrogen). All media were supplemented with 10% FBS and 1% penicillin-streptomycin (Gibco BRL). CCA60, CCA17, CCA55 and CCA21 were derived from patient tumors. Briefly, tumor cells were dissociated from primary tumors using collagenase, seeded and maintained in DMEM media. All the cell lines were maintained at 37℃ in 5% CO_2_ incubator. Mycoplasma contamination in cell culture was routinely tested every month. Commercial cells used for experiments were between 3 and 30 passages. PDC cells used for experiments were between 3 and 10 passages. Resistant cell EGI-1 R were was grown with increasing concentrations of gemcitabine, and the same duration passaged parental cells (EGI-1 P) were used in various cellular assays.

Human tissue samples were obtained from Sun Yat-sen University Cancer Center (Guangzhou, China) using protocols approved by the Institutional Review Board committee (G2023-136-01). Tumor tissue was examined by a pathologist to determine the tumor type and grade. Written informed consent was obtained from each individual who provided the tissue and all procedures were conducted in accordance with the medical ethical guidelines. The characteristics of these patients are listed in Supplementary Table S1.

CCA PDO(ATCC) was grown in organoid growth kit (ATCC, ACS-7101) and Matrigel. The PDOs were treated with azacitdine and olaparib and proliferation was assayed using CCK8 (TargetMol, C0005). Bliss synergy score was calculated using SynergyFinder 3.0.

Other reagents were purchased as follows: decitabine, azacitidine, olaparib and talazoparib were purchased from Target Mol (Shanghai, China). Kinase inhibitor drug library was obtained from Selleck Chemicals. Stock solutions were diluted in DMSO and stored according to the manufacturer's protocols.

### Colony formation, cell viability assay and combination index analysis

For the colony formation assay, single-cell suspensions were plated in 12 well plates (5,000 cells/well) or 6 well plates (10,000 cells/well) and incubated with indicated compounds for 8 to 10 days until the control group reached 80%-100% confluence. Colonies were fixed in methanol, stained with crystal violet (0.5% crystal violet, 20% methanol) and photographed.

For cell viability assay, cells were seeded in 96-well plates at the optimal seeding density (1000 cells/well) in triplicates. After 24 hours, cells were treated with different concentrations of drugs and cultured at 37℃ for 96 hours, and the number of viable cells was measured by using CellTiter-Glo reagent (#G7573, Promega). IC50 values were generated using GraphPad Prism 8. For combination index analysis, combination index values were determined by the inhibition rate of the cells and calculated using CalcuSyn software.

### Combinatorial drug screening

EGI-1 cells were subjected to a combinatorial drug screen with a kinase drug library. One thousand cells were seeded per well into 96-well plates and treated with compounds in the drug screen in the absence or presence of decitabine for 96 hours. Cell viability was assessed using CellTiter-Glo reagent according to the manufacturer's instructions. Differential drug sensitivity was determined by the C/S score, which is defined as control-normalized viability of decitabine and drugs library(combination) divided by control-normalized viability of drugs library (single). The results are listed in Supplementary Table S2.

### Gel-based semi-quantitative RT-PCR and qRT-PCR

Total RNA was extracted from cells using RNeasy Mini Kit (QIAGEN, Germany). Reverse transcription and quantitative PCR assays were performed using Trans Script All-in-OneFirst-Strand cDNA Synthesis SuperMix for qPCR (Transgene Biotech) and KAPA SYBR FAST qPCR Master Mix (2X) kit (Sigma-Aldrich). For the quantification of mRNA levels, 18s rRNA or GAPDH level was used as an internal control. For Gel-based semi-quantitative RT-PCR, the PCR products were visualized by 1% DNA agarose gel. The primer sequences are shown in Supplementary Table S3.

### Genomic DNA extraction, methylation-specific PCR and dot blot analysis

Genomic DNA was extracted from cell pellets according to the recommended protocol from the QIAamp® DNA Mini Kit (QIAGEN). Bisulfite modification of DNA was performed by using the EZ DNA methylation-Gold kit (ZYMO Research) according the manufacturer's instructions. MSP were performed in a 12.5 μl reaction mixture consisting of 0.6 μM of each primers targeting specific DNA promoters (Supplementary Table S3), 0.2 mM dNTP, 1.5 mM MgCl_2_, 1×PCR buffer, 0.5unit Gold-Taq (Applied Biosystems) and 0.5μl of bisulfited template DNA. The PCR products were analyzed on 2.0% agarose gels.

For dot blot analysis, DNA was extracted from cells using a TIANamp Genomic DNA Kit (Tiangen) according to the manufacturer's instructions. DNA was sonicated to generate fragments between 200 and 500 bp. Fragmented genomic DNA was diluted to 50 ng/μl in 20 μl of nuclease-free water. Then, DNA was incubated at 95 °C for 10 min and the tube was immediately placed on ice for 5 min. Then, 2 ul of the final DNA solution was added to separate wells of the 96-well dot blot apparatus. The nylon membrane was removed from the 96-well dot blot apparatus, dried at 60 °C for one hour, and crosslinked by UV irradiation at 1200 J/m2. The subsequent procedures were performed in accordance with the protocol for immunoblotting analysis.

### Flow cytometric analysis

Cell-cycle analysis was done by propidium iodide (PI, Sigma-Aldrich) staining to quantify the cell cycle phase. Briefly, 100,000 cells were seeded in 6-well plate. 24 hours later, cells were treated with indicated agents for 72 hours. Cells were fixed with 70% ethanol and stained with PI (50 mg/mL). To detect ROS in cells, harvested cells were washed once with PBS and incubated with PBS containing H2DCFDA (MedChemExpress, China) at 37 °C for 30 min in the dark. To detect the expression of MHC-I on the cell surface, cells were harvested via centrifugation (800 × g) at 4 °C for 5 min and incubated with anti-MHC class I polypeptide-related sequence (MIC)-A/B-FITC (cat. no. 53-5788-42) for 30 min at room temperature. The stained cells were analyzed by Ceytoflex SP6800 Spectral CellAnalyzer (Sony) and quantified by using the FlowJo software.

### Senescence-Associated β-Galactosidase Staining

β-Galactosidase activity in the cells was assessed using the Histochemical Staining kit (Servicebio cat: G1073-100T). The detection of β-galactosidase was performed following the manufacturer's instructions.

### Immunoblotting analysis

Protein extracts were prepared with RIPA cell lysis buffer (150 mM NaCl, 50 mM Tris-HCl, 0.5% deoxychlorate sodium, 200 mM NaF, 200 mM PMSF, 1.0% NP40, 1 mM EDTA) with the protease inhibitor cocktail (Roche, Basel, Switzerland). Lysates were subjected to SDS-PAGE and transferred to PVDF membrane for immunoblotting analysis. After blocking in 5% BSA (Sigma-Aldrich) and incubation with appropriate primary antibodies and secondary antibodies, immunoblotting was developed with ECL Western Blotting Detection Reagents (GE Healthcare Life Sciences) and detected with a Bio-Rad ChemiDoc MP imaging system. Antibodies used in this study were listed in Supplementary Table S4.

### Immunohistochemical staining

IHC staining was performed using standard procedures. Briefly, xenograft tumors were harvested, fixed with formalin, and embedded in paraffin. After deparaffinization, rehydration, antigen retrieval by heat-induced epitope retrieval, and inactivation of endogenous peroxidase by 3% H_2_O_2_, slides were blocked using a blocking solution and incubated overnight with primary antibodies. After incubation of secondary antibodies for 30 minutes, the DAB reagent kit (ZSGB-BIO, ZLI9019) was used as chromogen and hematoxylin (ZSGB-BIO, ZLI-9609) as counterstain. Antibodies used in this study were listed in Supplementary Table S4.

### Immunofluorescence staining

Cells were plated on 12 mm glass coverslips and incubated overnight for attachment. After the indicated treatments, the cells were fixed for 15 min at room temperature in 4% formaldehyde with 0.5% Triton X-100. Primary antibodies were incubated over night at 4 ºC. Secondary antibodies Molecular probes (Invitrogen) and DAPI (1 μg/ml) were incubated for 2 hours at room temperature. Coverslips were mounted using ProLong Gold (Invitrogen).

### Comet assay

Cells were mixed with LMA agarose and spread on comet slides. After 4°C solidification, the slides transferred into pre-chilled alkaline lysis solution (2.5 M NaCl, 100 mM Na_2_EDTA, 10 mM Tris-base, 10% DMSO, 1% Triton X-100, pH 10) for 2 hours at 4 °C. A denaturation step was performed in alkaline solution (300 mM NaOH, 1 mM EDTA, pH > 13) at room temperature for 20 min in the dark. The slide was then transferred to pre-chilled alkaline electrophoresis solution pH > 13 (300 mM NaOH, 1mM EDTA) and subjected to electrophoresis at 25 V, 300 mA for 30 min in the dark at 4 °C. Following electrophoresis, the slide was stained with propidium iodide solution and analyzed using a fluorescence microscope (Olympus, Japan).

### Chromatin retention assay and immunoblotting

The detailed procedure of immunoblotting was described in a previous publication 22. Antibodies used in this study were listed in Supplementary Table S4.

### RNA-sequencing

EGI-1 cells were seeded in six-well plates and treated with vehicle, 500 nM decitabine, 200 nM talazoparib, and their combination for 72 hours. Total RNA was extracted using the RNAeasy Mini Kit as the manufacturer's protocol (QIAGEN, Duesseldorf, Germany). The mRNA libraries were prepared using TruSeq Stranded Total RNA Sample Preparation kit with Ribo Zero Gold (Illumina), followed by sequencing on NovaSeq sequencer (Illumina). For RNA-seq analyses, raw counts were filtered and trimmed by fastp version 0.12.5 for clean data with default settings. The clean data were mapped to human reference genome (GRCh38, hg38) using STAR aligner (version 2.7.0f) and abundance of transcripts were quantified by RSEM 23, 24. Differential expression analyses were performed using the DEseq2 (v3.14.0) in the R statistical environment (v3.5). All further analyses were performed using R statistical programming.

### Animal experiments

Animal studies were conducted in compliance with animal protocols approved by the Institutional Animal Care and Use Committee of Sun Yat-sen University (SYSU-IACUC-2023-000322/SYSU-IACUC-2024-000955/SYSU-IACUC-2024-000894). Female BALB/c nude mice, NOD/SCID mice and C57BL/6 mice were purchased from Beijing Vital River Laboratory Animal Technology Company (Beijing, China) and housed under specific pathogen-free conditions in the Laboratory Animal Center of Sun Yat-sen University. Tumor volume was measured by vernier caliper and calculated with the following formula: tumor volume = width^2^ × length × 0.5237. Randomization was performed by equal division of tumor-bearing mice of similar tumor burden into different groups for drug treatment. For the EGI-1 tumor xenograft experiment, 3×10^6^ EGI-1 cells were injected subcutaneously. in the right flank of the BALB/c nude mice. For PDX mouse models, PDX-CCA17 tumor masses were passaged to NOD/SCID mice. When the tumors reached approximately 100 mm^3^, the mice were randomly divided into 4 groups for treatment: (a) vehicle; (b) decitabine (0.25mg/kg, every day, intraperitoneally); (c) talazoparib (0.3mg/kg, every day, oral gavage); and (d) combination (decitabine and talazoparib). Talazoparib was prepared weekly in 10% DMAc, 6% Solutol, and 84% PBS. Decitabine was dissolved in sterile PBS. The tumor volumes and body weights were monitored 3 times per week until tumor volume reached 1000-1500 mm^3^. These mice were sacrificed by CO_2_ inhalation, and their tumors were harvested for further analysis. To generate spontaneous murine CCA models, plasmids were introduced into mice through hydrodynamic tail-vein injection 25, 26. The amount of DNA into each mouse was 20ug pT3-EF1a-HA-myr-AKT, 30ug pT3-EF1a-YapS127A and 2.85ug SB transposase. The injection solution was administered into the tail vein of 7-week-old female C57BL/6 mice within 5 to 7 seconds. After three weeks, mice were randomly assigned to four treatment groups: vehicle, decitabine (0.25mg/kg, every day, intraperitoneally), talazoparib (0.3mg/kg, every day, oral gavage) or a combination of both drugs. All mice were killed after 2 weeks of drug treatment. Liver weight of each mouse was measured at the endpoint.

### Statistical analysis

Data are presented as mean ± SD/SEM. All statistical analyses were performed using GraphPad Prism version 8.0. Statistical differences were calculated using 2-tailed, unpaired Student's t test, 1-way ANOVA with Bonferroni's post hoc test, and 2-way ANOVA with Tukey's post hoc test or Fisher's exact test. In all statistical tests, statistical significance was considered as *P* < 0.05 unless stated otherwise.

### Accession Number

RNA-sequencing data were deposited in Gene Expression Omnibus with accession number GSE275134.

## Results

### *In vitro* effect of DNMT inhibitors azacitidine and decitabine in CCA cancer cells

**Figure 1:**
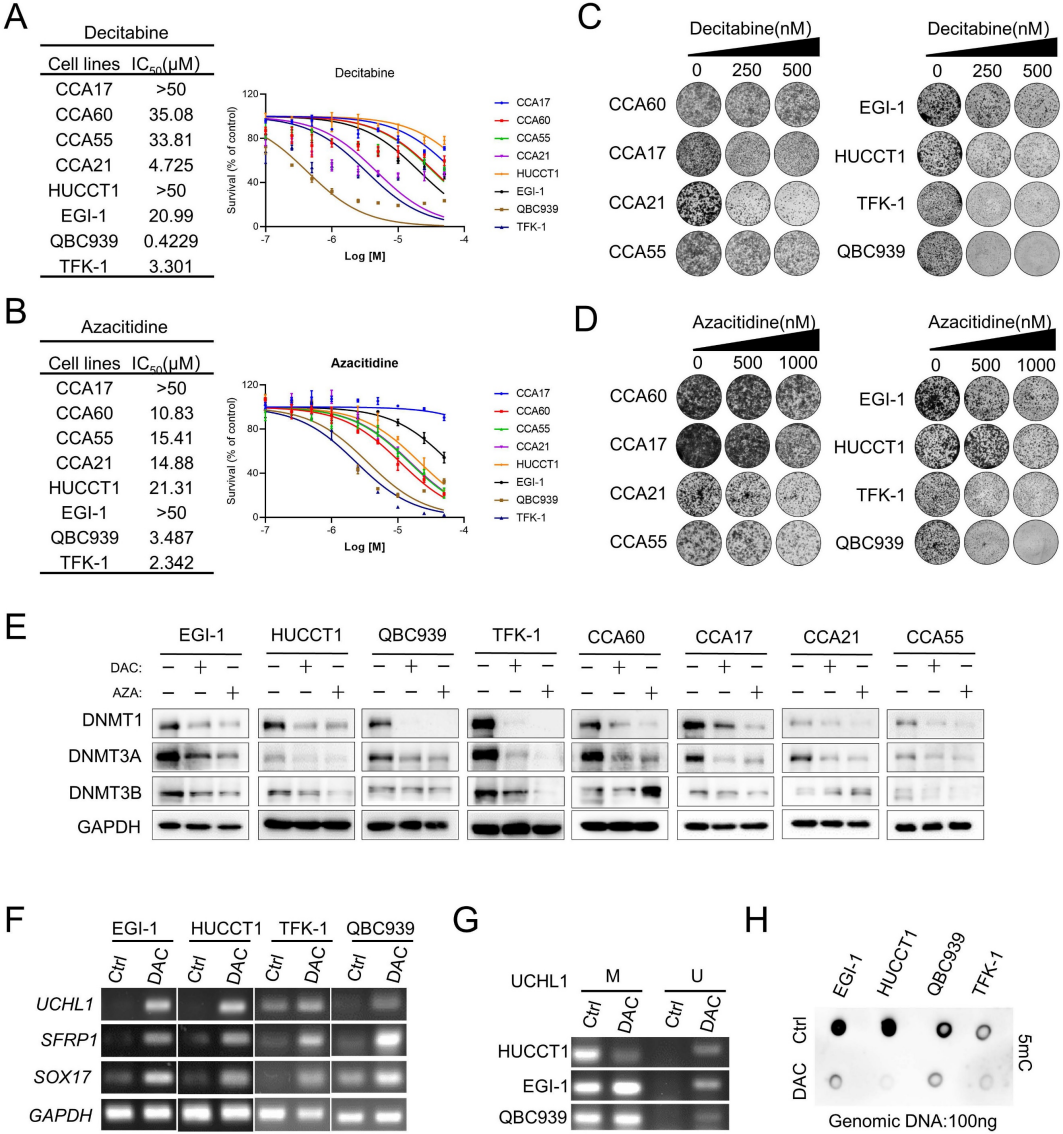
***In vitro* effect of decitabine and azacitidine in CCA Cancer Cells. (**A and B) CCA cells were treated with the indicated concentrations of decitabine(A) and azacitidine(B). The number of viable cells was measured at 96 hours. IC50 was calculated using GraphPad Prism software. (C and D) Colony formation assay of the indicated CCA cells treated with vehicle or decitabine and azacitidine. (E) Immunoblotting analysis of DNMT1, DNMT3A and DNMT3B in CCA cell lines treated with decitabine (DAC) and azacitidine (AZA) at the same dosage for 72 h. (F) RT-PCR analysis of *UCHL1*,* SFRP1* and *SOX17* in CCA cells treated with decitabine. (G) MSP analysis of *UCHL1* in EGI-1, HUCCT1 and QBC939 cells. M, methylated; U, unmethylated. (H) Indicated CCA cells were treated with decitabine for three days, followed by genomic DNA extraction and measurement of the levels of 5mC by dot blot.

To evaluate the effects of two FDA-approved nucleosides DMNTis in CCA, we established four patient-derived CCA cancer cells CCA17, CCA60, CCA21 and CCA55 **(Figure S1A)** as preclinical models and used four commercial CCA cell lines EGI-1, HUCCT1, TFK-1 and QBC939 to evaluate sensitivity to decitabine and azacitidine. Half-maximal growth-inhibitory concentration (IC50) analysis showed that the commercial CCA cell lines TFK-1 and QBC939 were relatively more sensitive, but the other CCA cell lines were relatively resistant to DNMTis, and most of them had complete resistance up to 10 μM **(Figure 1A and 1B)**. Similar findings were also observed in a previous study 27 and summarized here **(Figure S1B).** This unsatisfactorily inhibitory effect of DNMTi on cell growth was further confirmed by colony formation assays **(Figure 1C and 1D)**. Decitabine and azacitidine induce widespread genomic methylation changes by incorporating into DNA as a base and promoting DNMT degradation 28. To further determine whether DNA demethylation capacity in different cell lines plays a role in mediating the response to DNMTis, we firstly measured the protein levels of DNMT. The protein levels of DNMT1 and DNMT3A were significantly downregulated across all cell lines examined, whereas the protein level of DNMT3B was suppressed in some CCA cell lines **(Figure 1E)**. Increasing evidence suggests that DNMT1 protein levels could be used as a predictive biomarker to evaluate the pharmacological activity of DNMTis 29, 30. This observation raised the possibility that growth inhibitory of DNMTis is not related to the ability to demethylate DNA. In addition, decitabine treatment significantly increased the mRNA levels of *SFRP1*, *SOX17* and *UCHL1* in both sensitive and resistant cells (**Figure 1F**). Previous studies have shown that the expression of *SFRP1* and *SOX17* was regulated by the hypermethylated promoter state in CCA 31-33, but the hypermethylated promoter state of *UCHL1* has not been reported in CCA yet. Consistent with the restoration of *UCHL*1 expression, Methylation-specific PCR (MSP) data showed the demethylated promoter by decitabine in EGI-1, HUCCT1 and QBC939 cells (**Figure 1G**), suggesting that DNMTi activates the expression of silenced genes at low doses. Furthermore, we found that the overall DNA methylation level as detected by 5mC antibody decreased in both sensitive and resistant cells (**Figure 1H**), indicating that the DNA demethylation ability of DNMTis may not be enough to achieve the anti-tumor effects in CCA. These results suggest that alternative mechanisms may improve the anti-tumor efficacy of DNMTis in CCA.

### Combination drug screening identified PARP inhibitor as a sensitizer to DNMT inhibitor in CCA cancer cells and CCA organoid models

**Figure 2:**
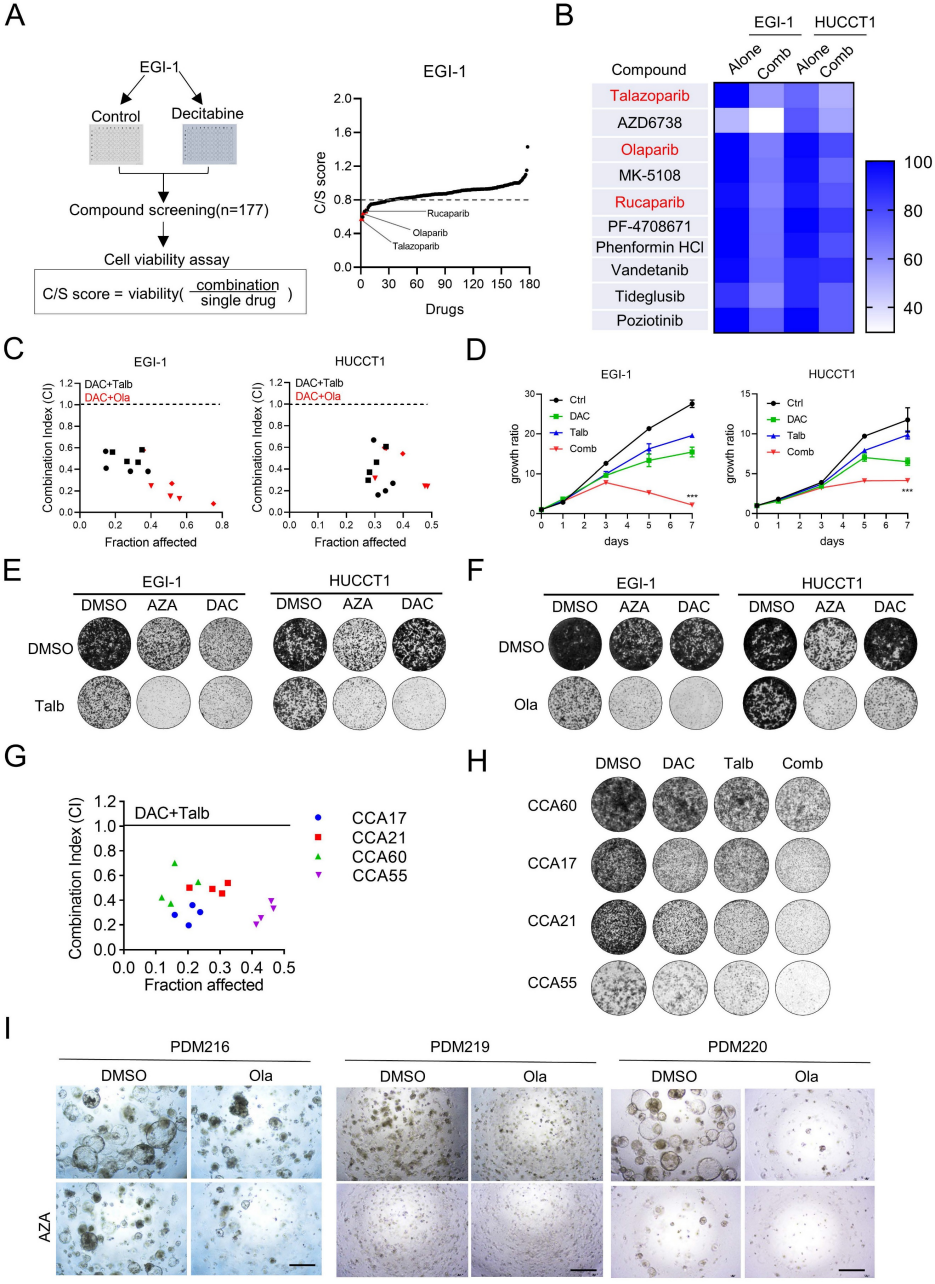
** PARP inhibitor potentiates anti-proliferation of decitabine in CCA cells and organoids *ex vivo*.** (A) The outline of drug-screening procedure. EGI-1 cells were seeded in 96-well plates and treated with the drug library in the presence or absence of decitabine during the primary drug screening. Right Graph showing the results of the drug-screening. Drugs on-screen are ranked according to the C/S (combination/single) score. Arrows highlight PARP inhibitors (red). (B) Heatmap indicates the cell viability of EGI-1 and HUCCT1 cells treat with the top selected 10 compounds from the drug-screening. (C) Drug combination index (CI) between decitabine and talazoparib/olaparib in EGI-1 and HUCCT1 cells based on 96h cell survival assay. CI values were assessed with CalcuSyn software (CI < 1, synergism; CI = 1, additive; CI > 1, antagonism). (D) The growth curve of EGI-1 and HUCCT1 cells with decitabine combined with talazoparib of indicated time points. (E-F) Representative images of colony formation assay in EGI-1 and HUCCT1 cells treated with vehicle, nucleoside DNMTi (decitabine and azacitidine), PARP inhibitor (talazoparib and olaparib) or their combination. (G) CI of decitabine and talazoparib in CCA PDC cells based on 96h cell survival assay. (H) Colony formation assay of CCA PDC cells treated with vehicle, decitabine, talazoparib or their combination. (I) Relative proliferation assay of CCA PDO treated with vehicle, azacitidine(AZA), olaparib(Ola) or their combination. Scale bar, 200 μm.

To explore the potential small molecules to improve the antitumor efficacy of DNMTi, we performed combinatorial drug screening with the kinase inhibitor library in resistant CCA cells EGI-1 to examine the effects of drug combination with decitabine (**Figure 2A**). After the first drug screening, the top ten kinase inhibitor candidates that showed good combinatorial effects were further confirmed in a second screening in another relatively resistant CCA cell HUCCT1. The results showed that PARP inhibitors (PARPis), including talazoparib, olaparib and rucaparib, were the most enriched among these kinase inhibitors and showed a combinatorial effect with decitabine compared to single treatment (**Figure 2B**).

The PARPis olaparib, rucaparib and talazoparib have received FDA approval for clinical use, primarily targeting the catalytic activity of PARP1 and PARP2 to suppress poly (ADP-ribose) polymerization and trap the PARP enzyme-DNA complex on single-strand breaks, leading to lethal double-strand breaks (DSBs) 34. Among these inhibitors, talazoparib exhibits superior therapeutic efficacy due to its enhanced PARP trapping capacity 35. To further determine whether DNMTis have a synergistic effect with PARPis, we performed a combination index analysis based on the Chou-Talalay combination index model in both EGI-1 and HUCCT1. We observed that all combination index values were less than 1.0, suggesting a synergistic effect between decitabine and PARPis (**Figure 2C**). The synergistic effects were further demonstrated by cell proliferation assays and colony formation assays (**Figure 2D-F**). We also found that the presence of this synthetic lethality effect in relatively sensitive cell lines QBC939 and TFK-1** (Figure S2A-B)**. Similar results were observed in patient-derived cells **(Figure 2G and 2H)**. In addition, we established acquired chemoresistance cell line (gemcitabine resistance) EGI-R** (Figure S2C)**. We demonstrated that combination treatment can inhibit the proliferation of EGI-R using cell viability assay and colony formation assay **(Figure S2D-E)**. These results suggest that the combination therapy can be used as a second-line clinical treatment for patients with chemoresistance. Similarly, in the CCA patient-derived organoid model (PDO), azacitidine and olaparib were more effective in combination than single treatment (**Figure 2I and Figure S2F**). Taken together, these results demonstrated that PARPis can indeed enhance the anti-tumor effect of nucleoside DNMTis in CCA cancer cells.

### The combination of decitabine and talazoparib enhanced anti-tumor effect in preclinical CCA models *in vivo*

**Figure 3:**
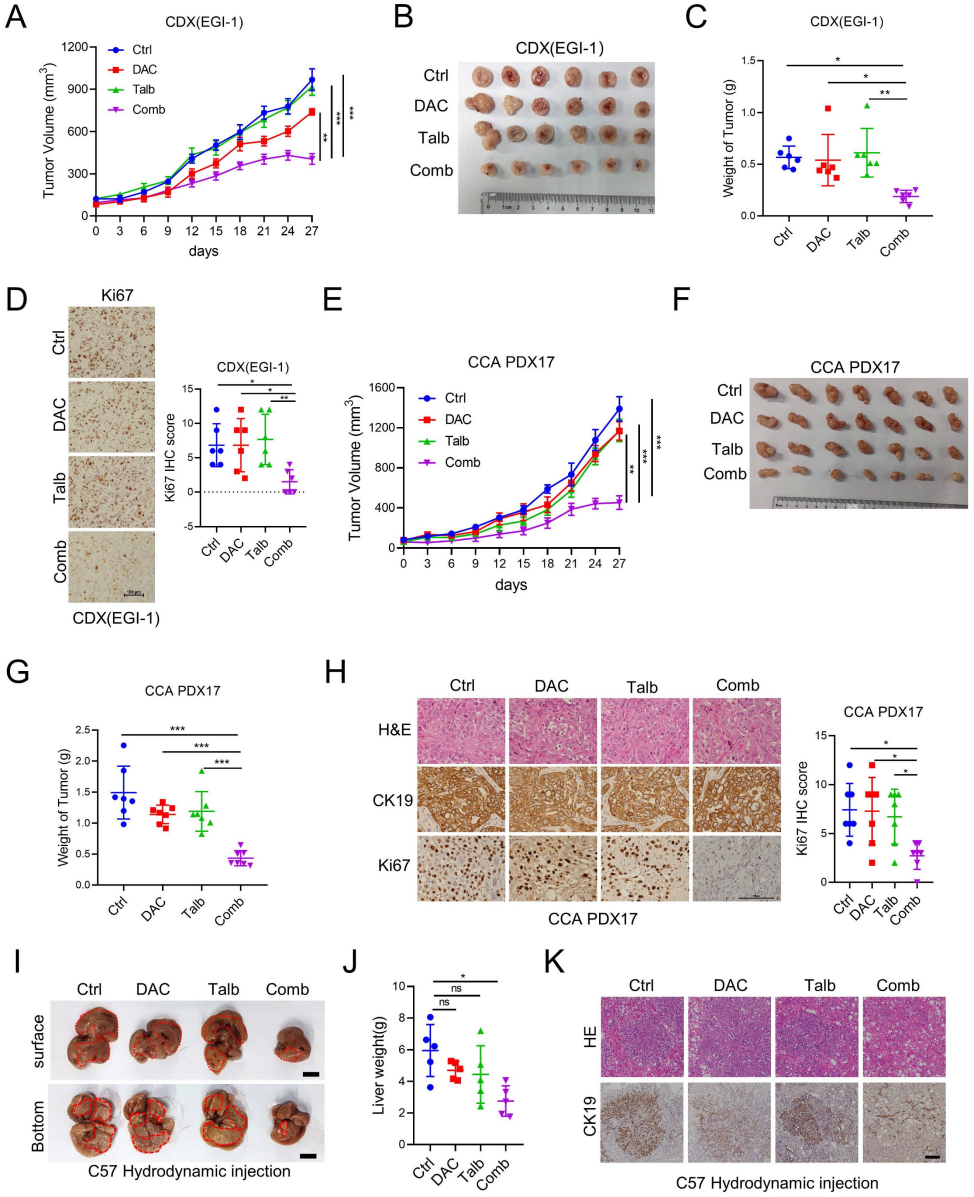
** The Combination of decitabine and talazoparib enhanced anti-tumor effects *in vivo*.** (A) Xenograft tumor growth curve of EGI-1 CCA cells in nude mice treated with decitabine, talazoparib or both. (B) The image shows the tumor sizes at the end of the xenograft tumor experiment. (C) Bar graph shows the tumor weight of (B). (D)Representative IHC and quantification of Ki67 in CDX EGI-1 of experiments described in A. Scale bar, 100μm. (E) Tumor growth curve of patient-derived xenograft CCA-PDX17 tumors in NSG mice treated with decitabine, talazoparib or both. (F) The image shows the tumor sizes at the end of the CCA-PDX17 experiment. (G) Bar graph shows the tumor weight of (E). (H) Representative IHC and quantification of Ki67 in PDX CCA17 of experiments described in E. Scale bar, 100 μm. (I) Representative images of liver tissues from the CCA orthotopic mouse model via hydrodynamic tail vein injection and then treated with decitabine, talazoparib or both for two weeks. Scale bar, 1 cm. (J) Liver weight were measured after the last dose of vehicle, decitabine, talazoparib or both. (K) Representative hematoxylin and eosin staining and IHC images of CK19 staining in CCA orthotopic mouse model of experiments described in D. Scale bar, 100 μm. (A and D) Date is presented as mean ± SEM. *P*-values were determined by two-way ANOVA with Tukey's post hoc test. (C, F and J) The data are presented as the mean ± S.D. *P* values were determined by 1-way ANOVA with Bonferroni's post hoc test. ns, not significant, *P < 0.05, **P < 0.01, ***P < 0.001.

To investigate the anti-tumor effect of the combination of decitabine and talazoparib *in vivo*, we injected EGI-1 cells into the flanks of SCID mice and treated the tumor-bearing mice with vehicle, decitabine, talazoparib, or the combination agents. The combination therapy significantly inhibited tumor growth compared to the monotherapy and control groups (**Figure 3A-C**). Decitabine, talazoparib or their combination treatment were well tolerated as demonstrated by maintenance of body weight in the treatment groups **(Figure S3A)**. IHC analysis of the xenograft tumors showed the significant decreased Ki-67 expression in combination treatment tumors compared to single treatment **(Figure 3D)**. To assess the clinical relevance of the above observations, we evaluated the synergistic effect of the combination in CCA patient-derived xenograft (PDX) models. Similar to our CDX model, the combination therapy effectively delayed tumor growth compared to talazoparib or decitabine alone (**Figure 3E-G**). The combination therapy effectively reduced tumor proliferation as indicated by decreased Ki-67 expression (**Figure 3H**). In addition, we examined combination therapy in a syngeneic mouse model developed by hydrodynamic tail vein injection 25, 26. The combination treatment resulted in a reduction in tumor burden compared to the control. Although the *p* value of liver weight was not statistically significant compared to single treatment, we observed a trend towards effective combination treatment **(Figure 3I-K)**. Taken together, these results support the notion that the combination of decitabine and talazoparib could enhance the antitumor effect in CCA *in vivo*.

### The combination treatment upregulated the inflammatory signaling pathways and suppressed the cell cycle-related pathways in CCA

**Figure 4:**
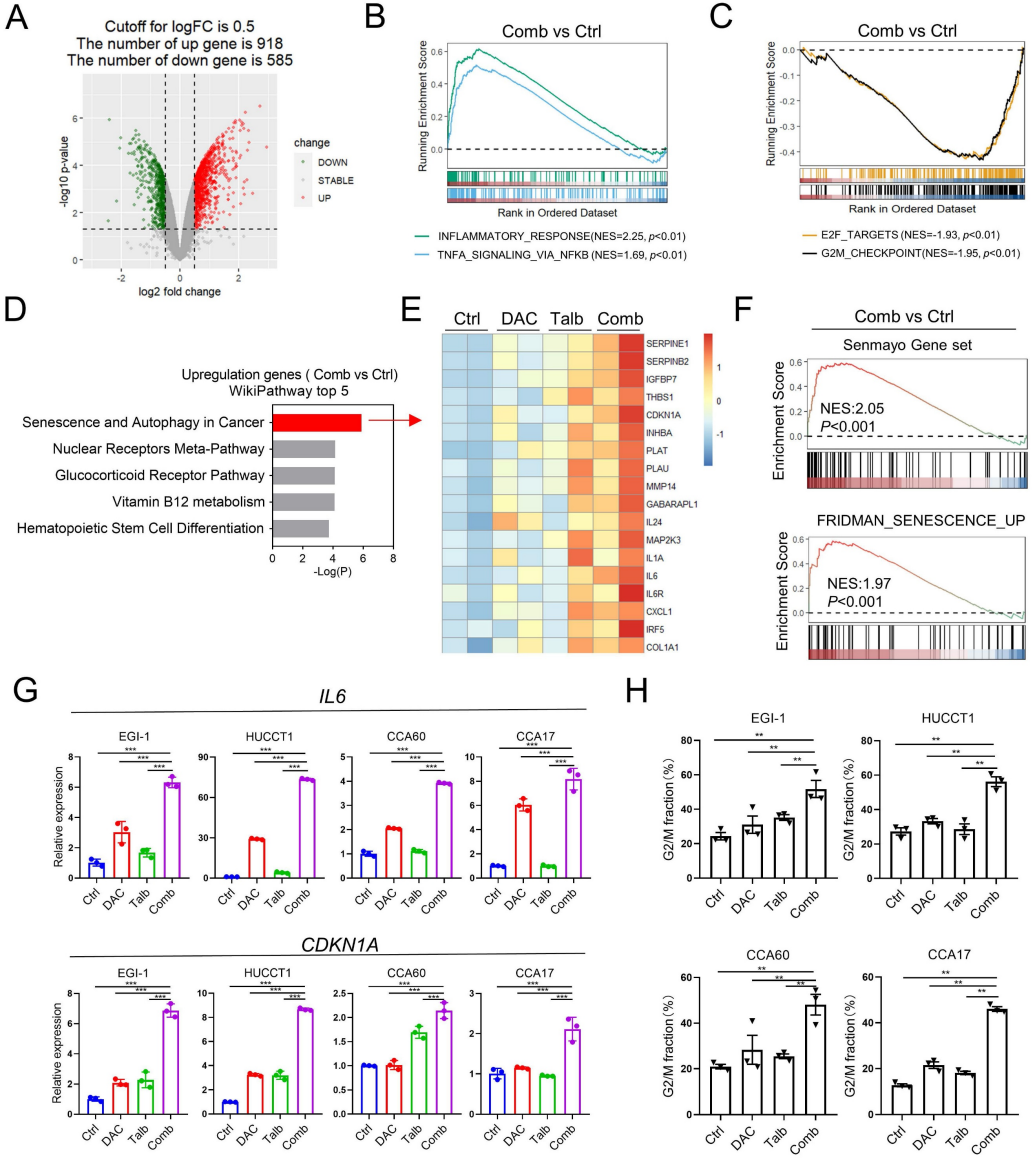
** Transcriptomic analysis indicated combination treatment affected inflammatory signaling pathway and cell cycle controlling pathway.** (A) The volcano map showing the differential expressed genes in EGI-1 treated with vehicle and the combination. (B and C) GSEA analysis of Hallmark pathway from EGI-1 cells treated with combined therapy compared with vehicle. (D) The WikiPathway analysis enriched by differentially expressed genes in the combination treatment relative to vehicle. (E) Heatmap of differential expressed genes in senescence and autophagy pathway under the treatment of decitabine, talazoparib and their combination. (F) GSEA analysis of indicated pathway from EGI-1 cells treated with combined therapy compared with vehicle. (G) The mRNA level of *IL6* and* CDKN1A* in EGI-1, HUCCT1, CCA60 and CCA17 cells treated with vehicle, decitabine, talazoparib and their combination for 72 h. (H) Cell cycle analysis of the indicated CCA cells treated with vehicle, decitabine, talazoparib and their combination for 72 h. The data are presented as the mean ± S.D. of three independent experiments. (G and H) *P*-values were determined by 1-way ANOVA with Dunnett's multiple comparisons test. ns, not significant, *P < 0.05, **P < 0.01, ***P < 0.001.

To investigate the underlying mechanisms of the synergistic effect of the combination treatment, we performed RNA-seq analysis to examine the transcriptional changes of EGI-1 treated with vehicle, decitabine, talazoparib, and their combination. The combination treatment resulted in a total of 1,503 differentially expressed genes, including 918 upregulated and 585 downregulated genes (**Figure 4A**). Gene set enrichment analysis (GSEA) showed that the most upregulated signaling pathways were mainly related to the inflammatory signaling pathways, including inflammatory response and TNF-alpha signaling via NF-κB (**Figure 4B and Figure S4A).** In addition, GSEA data indicated that the combination treatment also suppressed the cell cycle-related pathways, including G2M checkpoint and E2F targets (**Figure 4C and Figure S4A).** Furthermore, the WikiPathway analysis showed that senescence and autophagy signaling pathways were significantly enriched in cells treated with the drug combination (**Figure 4D-E**). Importantly, GSEA analysis indicated that other senescence-associated pathways were up-regulated in the combination group compared to control group in EGI-1 cells (**Figure 4F**). The upregulated genes involved in the senescence and autophagy pathways, including *IL6* and *CDKN1A,* were further validated in CCA cells (**Figure 4G**). We also confirmed that the combination treatment caused a significant change in cell cycle distribution with G2M arrest (**Figure 4H**).

Moreover, Gene Ontology (GO) pathway analysis revealed that the most downregulated pathways in combination treatment were correlated with genomic stability, such as the organization of nucleosome, the assembly of the protein-DNA complex and the protein localization in the centromeric region of the chromosome (**Figure S4B-D**). Collectively, these results demonstrated that the combination treatment caused cell cycle arrest and upregulation of inflammatory-related genes in CCA cells.

### PARP inhibitor combined with DNMTi Induced Cellular Senescence in CCA Cancer Cells

**Figure 5:**
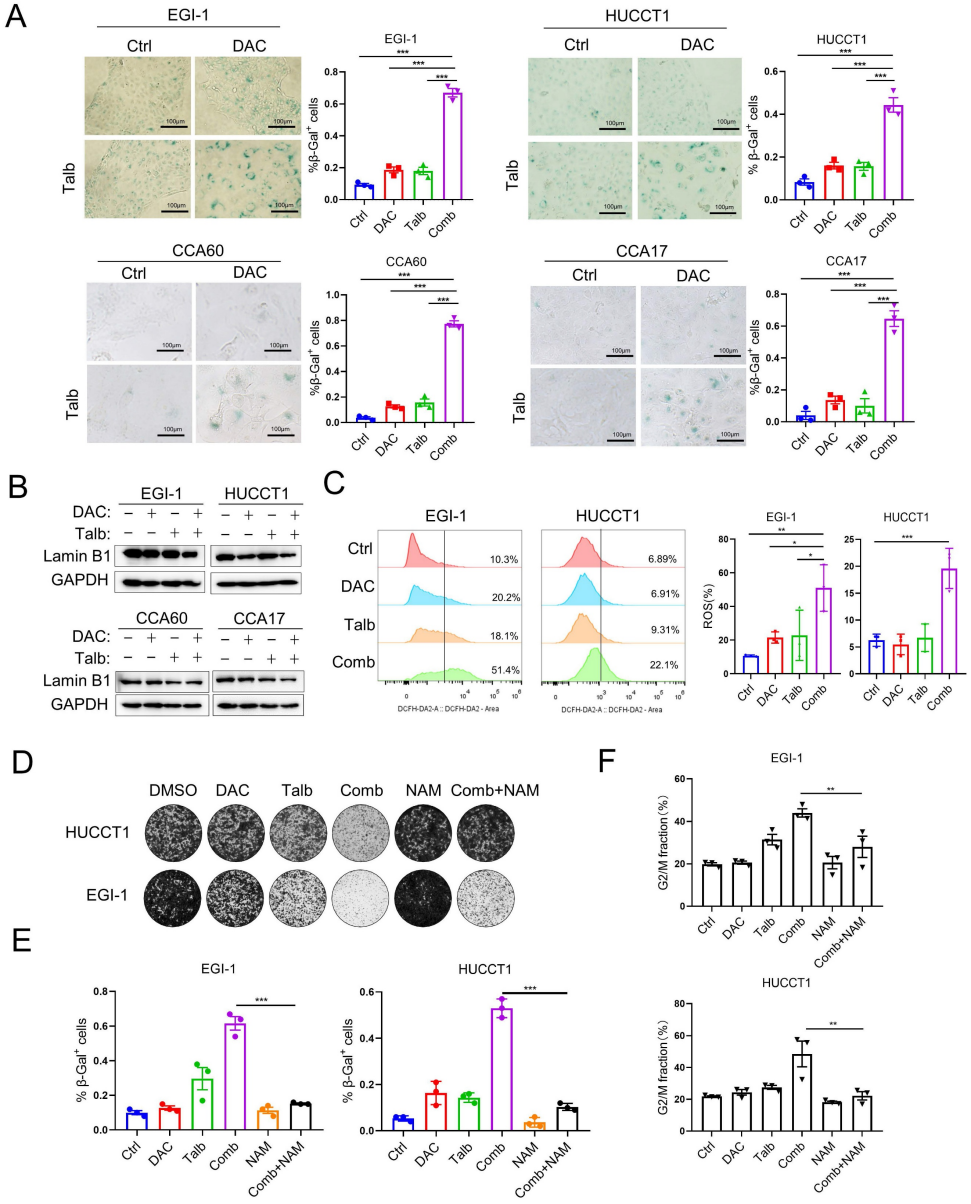
** Combined treatment induced cellular senescence in CCA.** (A) Representative SA-β-gal staining images and quantification of the indicated CCA cells treated with vehicle, decitabine, talazoparib and their combination for five days. (B) Immunoblotting analysis of laminB1 in CCA cells treated with vehicle, decitabine, talazoparib and their combination for 72 h. (C) Representative images and quantification of ROS level detection in EGI-1 and HUCCT1 treated with decitabine, talazoparib and their combination. (D) Colony formation assay of EGI-1 and HUCCT1 cells treated with vehicle, decitabine, talazoparib or their combination whereas the addition of 10 mM nicotinamide (NAM). (E) Quantification of the percentage of senescence cells that are SA-β-gal positive in different treatment. (F) Cell cycle analysis of cells treated with the combination with or without 10mM nicotinamide for 72 h. (A, C, E and F) *P*-values were determined by 1-way ANOVA with Dunnett's multiple comparisons test. ns, not significant; *P < 0.05, **P < 0.01, ***P < 0.001.

Senescent cells are characterized by stable cell cycle arrest and produce a senescence-associated secretory phenotype, evidenced by the secretion of proinflammatory cytokines, growth factors and matrix metalloproteinases 36. To determine whether the combination represents a potential therapeutic strategy to induce cellular senescence in CCA cells, we performed SA-β-Gal staining, the widely used cellular senescence marker, in CCA cells after drug treatment. The data showed that there were few cells stained with SA-β-Gal in the vehicle or single agent group, but most of the cells in the combination treatment were stained blue and showed senescence features by flattened and enlarged morphology (**Figure 5A**). In addition, the induction of senescence by the combination in CCA cells was further demonstrated by reduced laminB1 expression (**Figure 5B**). The senescence marker laminB1 was decreased and p21 was increased in CDX tumors and PDX tumors with combination treatment, suggesting that combination treatment induced significant cellular senescence (**Figure S5A**). Furthermore, common ROS detected by H2DCFDA was produced in large quantities in the combination treatment (**Figure 5C**). Senescent cells combine several properties that make them highly immunogenic and efficient in triggering adaptive antitumor immune responses 37. We next confirmed that MICA and MICB are more highly expressed on the cell surface upon combination treatment (**Figure S5B**), potentially leading to antitumor immune responses. Nicotinamide (NAM) is taken up by cells and readily converted into NAD^+^ via the salvage pathway, which is thought to improve cellular senescence and enhance anti-inflammatory properties 38-40. Interestingly, the combination induced cytotoxicity, whereas the addition of nicotinamide (NAM) can partially eliminate the combination-induced cell cytotoxicity (**Figure 5D**). Consistently, SA-β-gal staining analysis showed that the addition of NAM could partially rescue cellular senescence induced by the combination treatment (**Figure 5E**). Furthermore, the G2M phase arrest in the combination treatment can also recover by the addition of NAM (**Figure 5F**). Therefore, it is recommended to avoid concomitant use of nicotinamide in combination treatment of CCA and the combination of decitabine and talazoparib induced significant cellular senescence in CCA.

### Elevated levels of DNA damage induced senescence in the combination therapy to CCA

**Figure 6:**
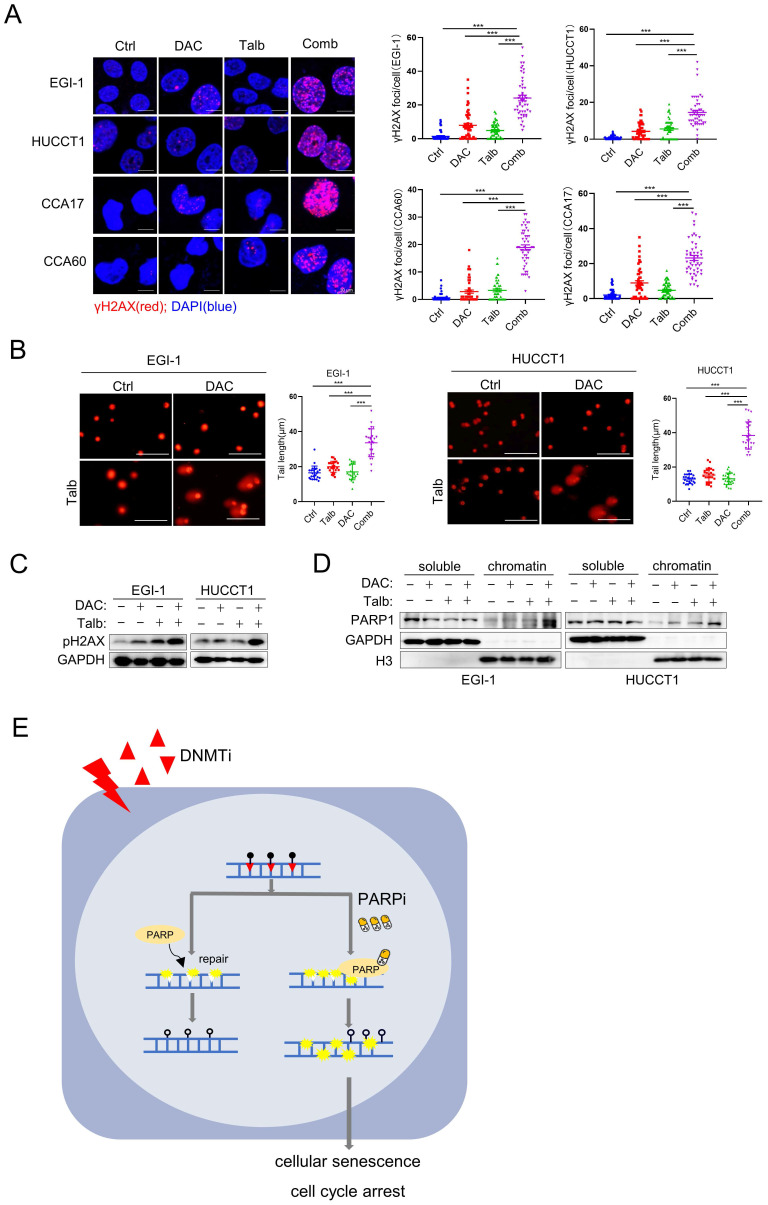
** Elevated levels of DNA damage in the combination therapy.** (A) Representative IF images of CCA cells stained with phosphorylated H2AX (γH2AX) and DAPI. Quantification of γH2AX foci in cells, n = 50(right). Scale bar, 10 μm.(B) Representative images of comet assay and quantification of comet tail length in EGI-1 and HUCCT1 cells treated with vehicle, decitabine, talazoparib, or their combination for 72 h. Scale bar, 100μm.* P*-values were determined by 1-way ANOVA with Dunnett's multiple comparisons test. ns, not significant; *P < 0.05, **P < 0.01, ***P < 0.001. (C) Immunoblotting analysis of indicated proteins in EGI-1 and HUCCT1 cells treated with vehicle, decitabine, talazoparib, or their combination for 72 h. (D) Levels of PARP1 in cell-equivalent aliquots of soluble and chromatin-containing fractions from EGI-1 and HUCCT1 following treatment for 24 h with vehicle, decitabine, talazoparib or their combination. (E) A schematic summary of the findings of this work.

Cellular senescence occurs in response to endogenous and exogenous stresses, including telomere dysfunction, oncogene activation, and persistent DNA damage 36. The nucleoside DNMTi is incorporated into DNA during replication, resulting in single-strand breaks (SSBs) 41, 42. PARP is an enzyme that plays a key role in the base excision repair pathway, particularly in the repair of SSBs. Recent reports have suggested that PARPis lead to the accumulation of unrepaired SSBs and eventually convert into DSBs by inhibiting the activity of PARP 43-45. GSEA analysis showed that downregulated genes are significantly enriched in the pathways involved in DNA damage-telomere stress-induced senescence, suggesting increased DNA damage in EGI-1 cells treated with the combination of drugs (**Figure S6A**). Based on these findings, we next determined whether the combination of decitabine with talazoparib could trigger DNA damage, which is one of the triggers of cellular senescence. By confocal immunofluorescence, we observed a significant increase in the number of DNA damage marker γH2AX foci after treatment with the combination of decitabine and talazoparib **(Figure 6A)**, indicating increased DNA damage. The protein level of γH2AX was also increased after the combination treatment** (Figure 6C)**. Similarly, the expression of the γH2AX was also increased in CDX and PDX tumors with combination treatment (**Figure S6B**). In addition, we observed a significant induction of DNA breaks by combination treatment in the neutral comet tail assay **(Figure 6B)**. PARPis are known for their potent PARP trapping ability, suggesting that it can more effectively trap PARP-DNA complexes. Consistent with previous study that the combination of DNMTis and PARPis could increase PARP trapping 46, we also observed that the combination treatment in CCA cells increased the trapping of toxic PARP (**Figure 6D**). Taken together, these results suggest that the combination of PARPi and decitabine may enhance the DNA damage and induce cellular senescence, thereby improving the efficacy of decitabine in CCA therapy as a therapeutic regimen.

## Discussion

Despite FDA-approved drugs for CCA, they target only a small subset of cases, and resistance to these drugs is widely reported. As a result, therapeutic options for CCA remain limited, presenting an urgent unmet clinical need.

Rather than relying on the long timelines and significant funding required for developing new drugs, we focus on identifying existing FDA-approved drugs that can exploit hypermethylation in CCA as a therapeutic target. These drugs could potentially be used in clinically more quickly, especially in compassionate care. We showed that PARPis can synergize with nucleoside DNMTis decitabine and azacitidine, in CCA. This combination of FDA-approved drugs enhances DNA damage and induces cellular senescence, offering a promising therapeutic strategy for CCA patients who do not respond to first-line chemotherapy. We demonstrated the effectiveness of the drug combination strategy in multiple preclinical CCA models *in vitro* and *in vivo*, including four patient-derived CCA cell lines and PDO. Both PARPis and DNMTis are already approved by FDA for other cancer indications such as myelodysplastic syndrome and acute myeloid leukemia. Although no similar large-scale trials of DNMTis or PARPis have been published in CCA, multiple prospective studies are underway, and their results are eagerly awaited. Therefore, our findings could easily be translated into clinical trials for CCA treatment.

Multi-omics studies had revealed profound in the DNA methylation landscape of CCA with concomitant transcriptome changes 10, 47. Transcriptional silencing of tumor suppressor genes through DNA hypermethylation was observed in CCA, leading to inhibition of cholangiocyte differentiation and activation of oncogenic pathways, including WNT, transforming growth factor-β, phosphoinositide 3-kinase, and NOTCH signaling pathways 32, 48, 49. In addition, several studies suggest that DNMTis could inhibit proliferation, migration and invasion of CCA cells through the reactivation of various tumor suppressor genes, such as apoptotic genes p53-BAX 50. In our study, we demonstrated that DNMTi activated the expression of genes silenced by DNA methylation at low dose, but was cytotoxic to CCA cells at high dosage. Therapeutic failure of DNMTis occurs in approximately 40% of patients 51, yet the resistance mechanisms remain incompletely elucidated. Emerging evidence implicates adaptive dysregulation of key metabolic pathways, including pyrimidine metabolism 30, 52, and genetic alterations ASXL1 as critical contributors to compromised treatment efficacy 51. This highlights those alternative strategies to enhance the therapeutic responses of DNMTi in cancers. Furthermore, clinical studies have shown that using high doses of decitabine or azacitidine can cause significant side effects, such as nausea, leucopenia, vomiting or decreased platelet count 53. Fortunately, lower dose of DNMTi may act synergistically with chemotherapy or immunotherapy 54, as shown in lymphoma 55, 56, where they have exhibited robust antitumor potency. Thus, DNMTis have been considered in combination with other cancer therapies and combination therapies could be a promising clinical strategy.

Our drug screening identified PARP inhibitor as a sensitizer to DNMTi in CCA cancer cells. Further studies demonstrated a synergistic effect between DNMTis and PARPis in both *in vitro* and *in vivo* CCA models, especially with patient-derived cells, organoid and xenograft models, providing strong preclinical evidence of its enhanced efficacy and low toxicity. Recent years have seen the FDA approval of many PARPis, including olaparib, niraparib, rucaparib and talazoparib in distinct settings. The use of PARP inhibitors has been indicted for both somatic or germline DNA repair gene mutations carriers, including BRCA1/2, ATM and so on 57. Previous study reported that DNMTis could promote homologous recombination deficiency by down-regulating key genes central to HR activity, including multiple genes in the Fanconi anemia (FA) pathway 58, which was not observed in our study. Here, our transcriptomic analysis demonstrated that the combination treatment upregulated the inflammatory signaling pathways, suppressed the cell cycle-related pathways and induced cellular senescence in CCA. Further investigation indicated that combination treatment caused severe DNA damage, which was an inducer of cellular senescence. In acute myeloid leukemia and breast cancer cells 46, low doses of DNMTis in combination with PARPis have been shown to increase the retention of PARP1 on chromatin through the induction of PARP1-DNA adducts and the covalent binding of DNMTs. This resulted in increased DNA damage, synergistic tumor cytotoxicity, attenuation of self-renewal and antitumor responses. Consistently, we also confirmed that PARP trapping increased in the combination treatment. Notably, the synergistic interaction between DNMTi and PARPi involves unique mechanistic complexity. Specifically, DNMTi exerts dual genotoxic effects: (1) Catalytic inhibition of DNMTs induces genome-wide hypomethylation, leading to chromatin structural remodeling and increased genomic instability; (2) During DNA replication, DNMTi incorporates into nascent strands as abnormal deoxycytidine analogs, generating single-strand DNA breaks 59. PARPis block the catalytic activity of PARP enzymes, thereby inhibiting the base excision repair pathway. This synthetic lethal interaction culminates in irreversible DNA damage accumulation, triggering cell cycle arrest and senescence **(Figure 6E)**. In addition, several studies showed that senescent cancer cells can be effectively eliminated by immune cells or senolytic compounds 60, 61. For example, senescent cells will upregulate the MHC-I-associated presentation machinery and IFN transcriptomic signature, which further activate autologous antigen-specific tumor-infiltrating CD8 lymphocytes 21. We also confirmed that cancer cells treated with the combination had high levels of immunogenic peptides on the cell surface, suggesting that combination therapy may activate T cell immunity. Therefore, our findings suggested that this drug combination could also be combined with immunotherapy to alter the tumor microenvironment to better eliminate CCA cells.

There are several limitations to our study, which should be addressed in future research. Long-term effects of this combination therapy, such as potential resistance mechanisms, need to be explored in greater detail. In-depth mechanisms mediating this combination effect, such as key hub genes or network, warrants further investigation. Furthermore, genetic mutations and epigenetic alterations in CCA may affect response to the combination drugs, and require more studies to better stratify patients who are likely to benefit from our proposed. Lastly, further investigation is needed to understand how the tumor microenvironment of CCA is affected by the drug combination, as this could influence the therapeutic response in clinical trials.

In summary, our study demonstrated the antitumor efficacy of the combination of DNMT inhibitors and PARP inhibitors in patient-derived CCA cell lines and patient-derived CCA xenograft models. Mechanistically, the combination treatment resulted in DNA damage and cellular senescence. Future clinical trials are needed to investigate the clinical effectiveness of this combination therapy in CCA.

## Supplementary Material

Supplementary figures and tables.

## Abbreviations

CCA: cholangiocarcinoma
DNMT: DNA methyltransferase
PARP: poly ADP-ribose polymerase
DNMTis: DNA methyltransferase inhibitors
DSBs: double-strand breaks
PARPis: PARP inhibitors
GO: Gene Ontology
DAC: decitabine
AZA: azacitidine
Ola: olaparib
Talb: talazoparib
PDO: patient-derived organoid
PDX: patient-derived xenograft
GSEA: Gene set enrichment analysis
HE: Hematoxylin-Eosin
IHC: Immunohistochemical
