# Supplementary material for: PARP inhibitor augments anti-tumor efficacy of DNMT inhibitor by inducing senescence in cholangiocarcinoma
Source: Int J Biol Sci. 2025 May 27;21(8):3649–65. doi: 10.7150/ijbs.110947 (PMC12160928; doi:10.7150/ijbs.110947)
Supplement: Supplementary file 1 — Supplementary figures and tables. [file ijbsv21p3649s1.pdf]

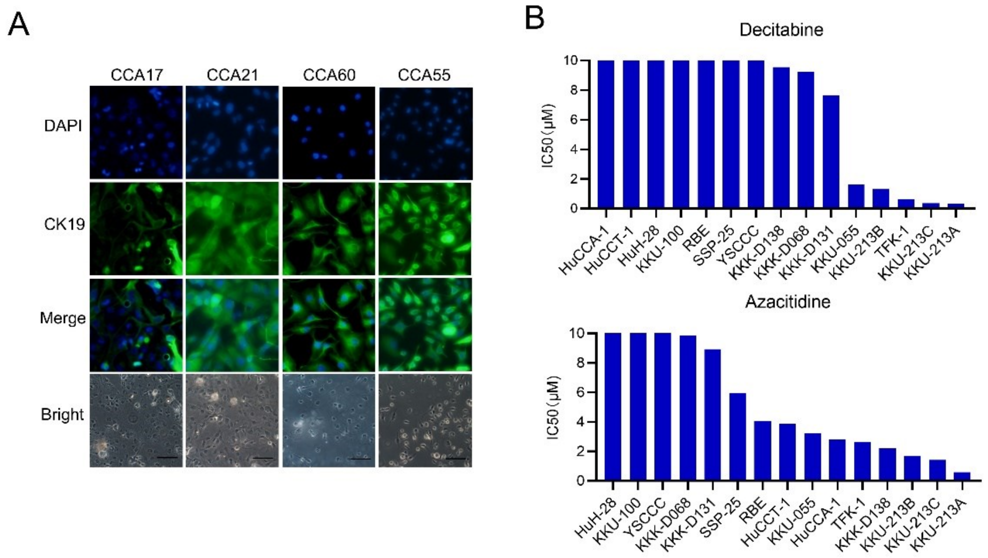

Supplementary Figure S1

(A) Representative IF images and bright images of CCA PDC cells stained with CK19 and DAPI. Scale bar, 50 μm. (B) The half-maximal inhibitory concentration (IC50) values for decitabine and azacitidine are represented on the y axis in commercial CCA cell lines from drug response data (Supawan Jamnongsong.etl).

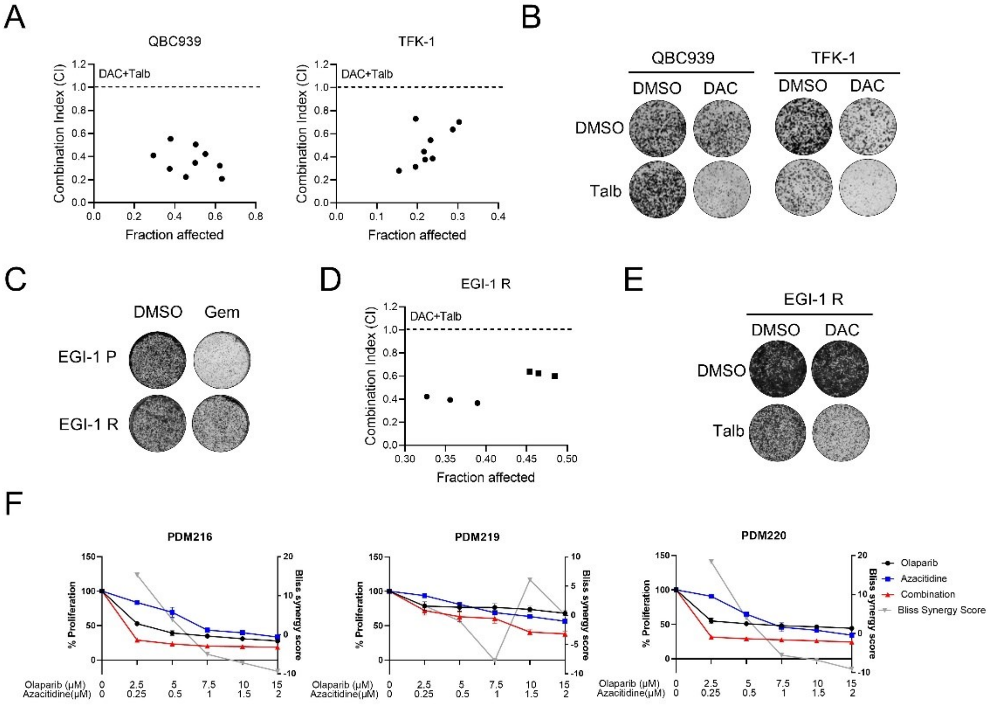

Supplementary Figure S2

(A) Drug combination index (CI) between decitabine and talazoparib in QBC939 and TFK-1 cells based on 96h cell survival assay. CI values were assessed with CalcuSyn software (CI < 1, synergism; CI = 1, additive; CI > 1, antagonism). (B) Representative images of colony formation assay in QBC939 and TFK-1 cells treated with vehicle, decitabine, talazoparib or their combination. (C) Colony formation assay of EGI-1 parental (P) and resistant (R) cells treated with vehicle and gemcitabine. (D) CI of decitabine and talazoparib in EGI-1 R cells based on 96 h cell survival assay. (E) Colony formation assay of EGI-1 R treated with vehicle, decitabine, talazoparib or their combination. (F) Relative proliferation assay of CCA PDO treated with vehicle, decitabine, talazoparib or their combination. The relative proliferation was determined using CCK8 assays. The synergy score was calculated as the deviation from the theoretical inhibition calculated by the Bliss method. Here, a synergy score of ± 10 % indicates an additive interaction, below -10 % an antagonistic interaction, and above 10 % synergy.

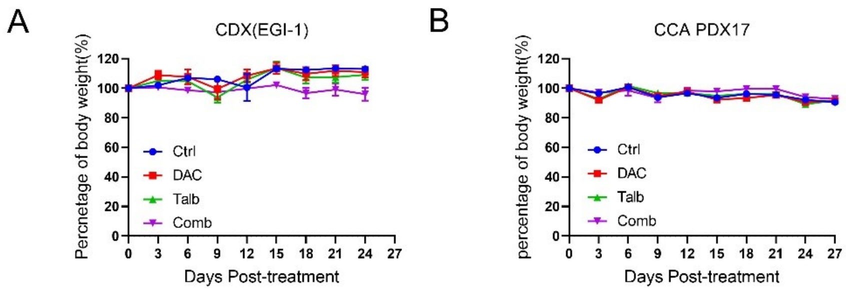

Supplementary Figure S3

(A and B) Mice weight of indicated group during the period of treatment

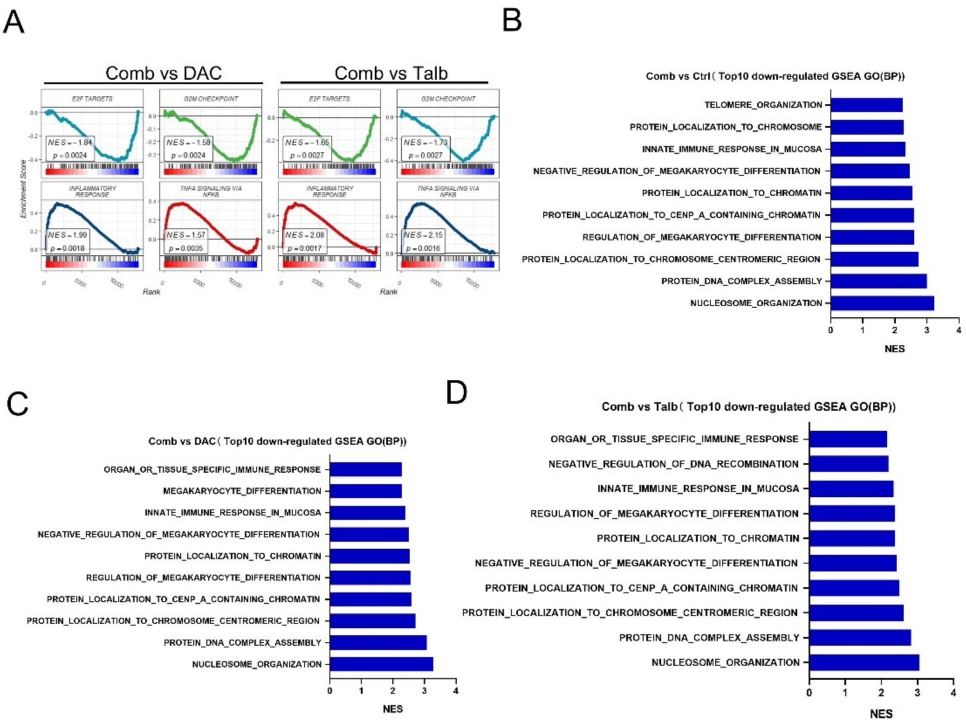

Supplementary Figure S4

(A) GSEA analysis of Hallmark pathway from EGI-1 cells treated with combined therapy compared with single drug treatments. (B-D) Normalized enrichment score (NES) graph of the top 10 down-regulated GSEA gene ontology (GO) Biological Process (BP) pathways of EGI-1 cell lines treated with combined therapy compared with control(B), decitabine(C) and talazoparib(D).

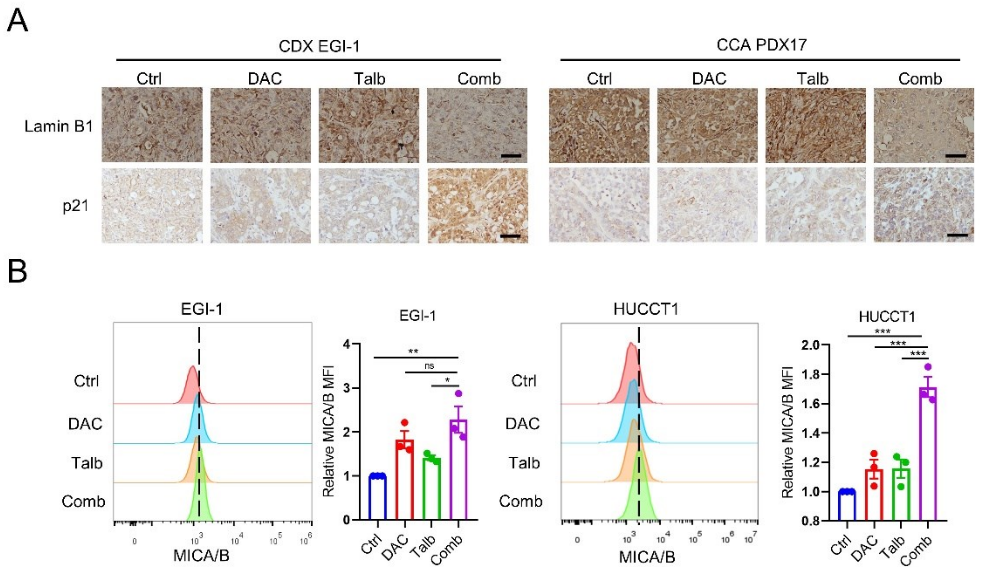

Supplementary Figure S5

(A) Representative images of Lamin B1 and p21 staining were performed on formalin-fixed, paraffin-embedded PDXs and CDXs after the last dose of vehicle, decitabine, talazoparib, or the combination treatment. Scale bar, 50 μm. (B) MICA/B expression in EGI-1 and HUCCT1 cells after decitabine, talazoparib, or the combination treatment.

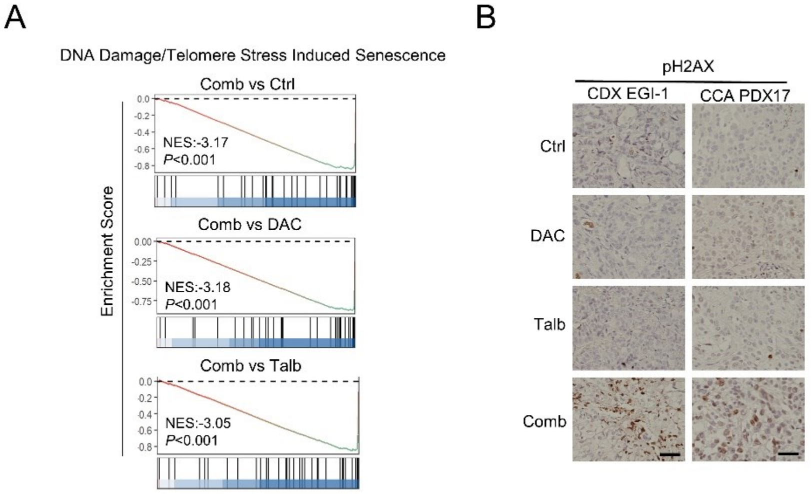

Supplementary Figure S6

(A) GSEA analysis of Reactome pathway from EGI-1 cells treated with combined therapy compared with vehicle showed that the indicated pathway was significantly enriched. NES, normalized enrichment score. (B) Representative images of pH2AX staining were performed on formalin-fixed, paraffin-embedded PDXs and CDXs after the last dose of vehicle, decitabine, talazoparib or the combination treatment. Scale bar, 50 μm.

Supplementary Table S1. Characteristics of Patients

patient ID	gender	age	Clinical Characteristics	Immunohistochemistry
CCA17/PD X 17	male	64	cholangiocarcinoma	CK(AE1/AE3) (+), CK7 (+), CEA (+), CDX2 (-), CK20 (-); #2D:CK7 (+), Muc-1 (+), Muc-5AC (-), CK20 (-), CDX2 (-), Muc-2 (-), SATB2 (weak +), HepPar-1(-), TTF-1 (-), PSA (-). in situ hybridization results: EBERs (-).
CCA21	male	70	cholangiocarcinoma	CK7 (+), CK19 (+), Muc-1(+), Muc-2 (-), CDX2 (-), HepPar-1(-), AFP (-), Arginase-1(-); in situ hybridization Arginase - 1 results: EBERs (-).
CCA60	female	65	cholangiocarcinoma	HepPar-1(-), AFP (Minor positivity), Arginase-1(-), Glypican-3(-), CK7(+), CK19(+), CD34(-), CD10(+), Muc-5AC (-).
CCA55	male	61	cholangiocarcinoma	HepPar-1(-), AFP (-), Arginase-1(-), Glypican-3(-), CK7(+), CK19(+), CD34(-), CD10(+).

Supplementary Table S2. Drug screening results

Index	Molname	C/S score
1	Talazoparib	0.580938
2	AZD6738	0.596867
3	Olaparib (AZD2281, Ku-0059436)	0.649823
4	MK-5108 (VX-689)	0.663753
5	Rucaparib (AG-014699,PF-01367338) phosphate	0.671304
6	PF-4708671	0.675687
7	Phenformin HCl	0.676951
8	Vandetanib (ZD6474)	0.711248
9	Tideglusib	0.728468
10	Poziotinib (HM781-36B)	0.736794
11	A-769662	0.74919
12	Selumetinib (AZD6244)	0.749787
13	ONO-4059 analogue	0.751781
14	GSK2636771	0.753358
15	AZ20	0.754478
16	Dasatinib	0.755809
17	CHIR-98014	0.757602
18	Voxtalisib (XL765, SAR245409)	0.761909
19	Veliparib (ABT-888)	0.763602
20	Vemurafenib (PLX4032, RG7204)	0.764828
21	MK-2206 2HCl	0.767207
22	Navitoclax (ABT-263)	0.770293
23	Anastrozole	0.773078
24	Ribociclib (LEE011)	0.775537
25	Ibrutinib (PCI-32765)	0.776217
26	Binimetinib (MEK162, ARRY-162, ARRY-438162)	0.778409
27	PX-478 2HCl	0.788141
28	Ceritinib (LDK378)	0.791767
29	NSC 319726	0.791804
30	BMS-777607	0.79198
31	Ridaforolimus (Deforolimus, MK-8669)	0.797731
32	ETP-46464	0.799121
33	Ulixertinib (BVD-523, VRT752271)	0.799812
34	Idelalisib (CAL-101, GS-1101)	0.800674
35	Trametinib (GSK1120212)	0.803234
36	AZD3463	0.803629
37	Quercetin	0.805562
38	Tivozanib (AV-951)	0.809509
39	Uprosertib (GSK2141795)	0.810655
40	LY2584702 Tosylate	0.813713
41	KX2-391	0.815508
42	Indirubin	0.816487

43	Triciribine	0.816816
44	BX-795	0.817017
45	BMS-536924	0.817147
46	MLN8054	0.817671
47	FG-2216	0.819743
48	ERK5-IN-1	0.822514
49	Rapamycin (Sirolimus)	0.823649
50	Picropodophyllin (PPP)	0.824405
51	PF-06463922	0.825478
52	NLG919	0.826892
53	WH-4-023	0.827126
54	Varlitinib	0.828797
55	Afatinib (BIBW2992) Dimaleate	0.829166
56	Bisindolylmaleimide I (GF109203X)	0.832816
57	Lenalidomide (CC-5013)	0.832993
58	TGX-221	0.833459
59	NSC348884	0.837655
60	Linifanib (ABT-869)	0.841446
61	YM155 (Sepantronium Bromide)	0.84267
62	Ponatinib (AP24534)	0.842712
63	Fostamatinib (R788)	0.843416
64	SC1	0.847353
65	Duvelisib (IPI-145, INK1197)	0.848206
66	Chloroquine Phosphate	0.851384
67	GSK690693	0.852868
68	Apremilast (CC-10004)	0.853036
69	SB203580	0.853362
70	Lapatinib (GW-572016) Ditosylate	0.853896
71	Buparlisib (BKM120, NVP-BKM120)	0.855046
72	Losmapimod (GW856553X)	0.8553
73	Linsitinib (OSI-906)	0.857367
74	Exemestane	0.858054
75	FR 180204	0.860456
76	AICAR (Acadesine)	0.862226
77	Flavopiridol (Alvocidib)	0.864056
78	Voxtalisib (SAR245409, XL765) Analogue	0.865119
79	Quizartinib (AC220)	0.867845
80	CNX-774	0.868309
81	ABC294640	0.868375
82	Doramapimod (BIRB 796)	0.868537
83	S-Ruxolitinib (INCB018424)	0.868788
84	GSK481	0.868854
85	Pazopanib HCl (GW786034 HCl)	0.868989
86	Pifithrin-μ	0.86975

87	Imatinib Mesylate (STI571)	0.869787
88	Cediranib (AZD2171)	0.871119
89	Pacritinib (SB1518)	0.871364
90	TWS119	0.871887
91	R406 (free base)	0.87205
92	Venetoclax (ABT-199, GDC-0199)	0.876291
93	Dacomitinib (PF299804, PF299)	0.880281
94	Lenvatinib (E7080)	0.881152
95	Epacadostat (INCB024360)	0.883109
96	Tofacitinib (CP-690550) Citrate	0.883693
97	BI-D1870	0.885669
98	Vistusertib (AZD2014)	0.886598
99	Masitinib (AB1010)	0.893771
100	Cabozantinib (XL184, BMS-907351)	0.894585
101	Sunitinib	0.896247
102	Sorafenib Tosylate	0.897083
103	Alpelisib (BYL719)	0.89965
104	Pomalidomide	0.900098
105	SB415286	0.903425
106	Alisertib (MLN8237)	0.903497
107	Everolimus (RAD001)	0.908236
108	Sotrastaurin	0.91236
109	Dinaciclib (SCH727965)	0.912613
110	VE-822	0.913424
111	Vatalanib (PTK787) 2HCl	0.913595
112	Crizotinib (PF-02341066)	0.916051
113	PHA-665752	0.916055
114	Fedratinib (SAR302503, TG101348)	0.916618
115	NVP-AEW541	0.916815
116	Pictilisib (GDC-0941)	0.916911
117	Tivantinib (ARQ 197)	0.917341
118	SANT-1	0.921518
119	WZ4003	0.922353
120	PF-04217903	0.923037
121	CHIR-99021 (CT99021)	0.924051
122	PD0325901	0.924054
123	SU11274	0.924204
124	SB202190 (FHPI)	0.924962
125	Saracatinib (AZD0530)	0.925266
126	Foretinib (GSK1363089)	0.925985
127	Rociletinib (CO-1686, AVL-301)	0.926325
128	abemaciclib (LY2835219)	0.92668
129	Go 6983	0.927385
130	NSC59984	0.927413

131	Dabrafenib (GSK2118436)	0.92765
132	Nilotinib (AMN-107)	0.929061
133	VX-11e	0.929272
134	Daprodustat (GSK1278863)	0.929616
135	Tofacitinib (CP-690550,Tasocitinib)	0.929855
136	Torin 2	0.930659
137	AT9283	0.932657
138	Momelotinib (CYT387)	0.933289
139	INCB024360 analogue	0.934625
140	AT7867	0.938494
141	JNJ-38877605	0.938637
142	Sorafenib	0.942435
143	PHT-427	0.942651
144	IOX2	0.942958
145	G-749	0.952286
146	Ozanimod (RPC1063)	0.953169
147	SGX-523	0.953468
148	KC7F2	0.95618
149	Go6976	0.958872
150	Axitinib	0.95967
151	Roxadustat (FG-4592)	0.961368
152	Danusertib (PHA-739358)	0.968074
153	XMD8-92	0.971502
154	Dovitinib (TKI-258, CHIR-258)	0.971813
155	Tozasertib (VX-680, MK-0457)	0.974876
156	Dovitinib (TKI258) Lactate	0.976769
157	Apitolisib (GDC-0980, RG7422)	0.98187
158	Radotinib	0.984561
159	Entrectinib (RXDX-101)	0.987338
160	GDC-0879	0.988254
161	Pazopanib	0.989114
162	Vismodegib (GDC-0449)	0.999147
163	Cerdulatinib (PRT062070, PRT2070)	1
164	DEL-22379	1
165	Roscovitine (Seliciclib,CYC202)	1
166	Refametinib (RDEA119, Bay 86-9766)	1
167	Ro 31-8220 Mesylate	1
168	Pilaralisib (XL147)	1.001426
169	PLX7904	1.015074
170	Osimertinib (AZD9291)	1.023406
171	SU6656	1.035642
172	TCS 359	1.055969
173	Schisandrin B (Sch B)	1.06543
174	Enzastaurin (LY317615)	1.079365

175	Afuresertib (GSK2110183)	1.099345
176	Omipalisib (GSK2126458, GSK458)	1.151426
177	GDC-0032	1.428858

Supplementary Table S3. q-PCR and MSP Primer sequence

Primers	forward primer (5-3)	reverse primer (5-3)
SOX17	CAGGTCTCGGACTACG CTG	CCGTAGTACACGTGAAGG GC
SFRP1	CAATGCCACCGAAGCC TCCAAG	CAAACTCGCTGGCACAGA GATG
UCHL1	CAGTTCAGAGGACACC CTGCTG	CCACAGAGCATTAGGCTG CCTT
18S	GGACACGGACAGGATT GACA	ACCCACGGAATCGAGAA AGA
CDKN1A	AGGTGGACCTGGAGAC TCTCAG	TCCTCTTGGAGAAGATCA GCCG
GAPDH	GTCTCCTCTGACTTCAA CAGCG	ACCACCCTGTTGCTGTAG CCAA
IL6	AGACAGCCACTCACCT CTTCAG	TTCTGCCAGTGCCTCTTT GCTG
UCHL1 MSP methylated primer	TTTATTTGGTCGCGATC GTTC	AAACTACATCTTCGCGAA ACG
UCHL1 MSP unmethylated primer	GGGTTTGTATTTATTTG GTTGT	CTTAAACTACATCTTCAC AAAACA

Supplementary Table S4. Antibodies used in this study

Antibodies	Source	Product No.
Phospho-Histone H2AX	CST	9718S
GAPDH	CST	2118S
DNMT1	ABclonal	A22455
laminB1	ABclonal	A1910
HRP-conjugated anti-rabbit	GE Healthcare Life Sciences	NA934
HRP-conjugated anti-mouse	GE Healthcare Life Sciences	NA931
Ki67	ZSGB-BIO	ZA-0502
CK19	proteinteh	10712-1-AP
CDKN1A/p21	ABclonal	A19094
5-Methylcytosine (5mC) Rabbit mAb	ABclonal	A20599
PARP	CST	9542S
Histone H3	CST	9715S
